# Development and Validation of Deep Learning Preoperative Planning Software for Automatic Lumbosacral Screw Selection Using Computed Tomography

**DOI:** 10.3390/bioengineering11111094

**Published:** 2024-10-30

**Authors:** Baodong Wang, Congying Zou, Xingyu Liu, Dong Liu, Yiling Zhang, Lei Zang

**Affiliations:** 1 Department of Orthopedics, Beijing Chao-Yang Hospital, Capital Medical University, Beijing 100043, China; wangbaodong@mail.ccmu.edu.cn (B.W.); cleverleyzou@mail.ccmu.edu.cn (C.Z.); 2 School of Life Sciences, Tsinghua University, Beijing 100084, China; xyliu@changmugu.com; 3 Institute of Biomedical and Health Engineering (iBHE), Tsinghua Shenzhen International Graduate School, Shenzhen 518000, China; 4 School of Biomedical Engineering, Tsinghua University, Beijing 100084, China; 5 Longwood Valley Medical Technology Co., Ltd., Beijing 101111, China; liudong@changmugu.com

**Keywords:** posterior lumbar interbody fusion, deep learning, computed tomography, pedicle screws, Gertzbein–Robbins classification, Badu grading

## Abstract

Achieving precise pedicle screw placement in posterior lumbar interbody fusion (PLIF) is essential but difficult due to the intricacies of manual preoperative planning with CT scans. We analyzed CT data from 316 PLIF patients, using Mimics software for manual planning by two surgeons. A deep learning model was trained on 228 patients and validated on 88 patients, assessing planning efficiency and accuracy. Automatic planning successfully segmented and placed screws in all 316 cases, significantly outperforming manual planning in speed. The Dice coefficient for segmentation accuracy was 0.95. The difference in mean pedicle transverse angle (PTA) and pedicle sagittal angle (PSA) for automatic planning screws compared to manual planning screws was 1.63 ± 0.83° and 1.39 ± 1.03°, respectively, and these differences were either statistically comparable or not significantly different compared to the variability of manual planning screws. The average Dice coefficient of implanted screws was 0.63 ± 0.08, and the consistency between automatic screws and manual reference screws was higher than that of internal screws (Dice 0.62 ± 0.09). Compared with manual screws, automatic screws were shorter (46.58 ± 3.09 mm) and thinner (6.24 ± 0.35 mm), and the difference was statistically significant. In qualitative validation, 97.7% of the automatic planning screws were rated Gertzbein–Robbins (GR) Class A and 97.3% of the automatic planning screws were rated Badu Class 0. Deep learning software automates lumbosacral pedicle screw planning, enhancing surgical efficiency and accuracy.

## 1. Introduction

Low back pain is a very common condition, and the number of patients suffering from low back pain has increased over the past 10 years. It is estimated that about 619 million people worldwide suffer from low back pain [1,2]. Degenerative lumbar diseases are the leading cause of low back pain and spinal dysfunction, resulting in a significant social and economic burden [3]. Posterior lumbar interbody fusion (PLIF) is the gold standard for treating this condition when conservative treatment fails [4]. However, PLIF is risky and complex, prone to nerve/blood vessel damage, steep learning curve, and radiation damage [5,6]. The success of PLIF heavily depends on the precise placement of pedicle screws. In addition, careful preoperative planning is essential to improve screw placement accuracy, making it necessary to plan the screw size and trajectory properly [7,8,9,10].

Pedicle screw preoperative planning software included traditional manual planning software, such as Mimics, 3D slicer, and Surgimap [11,12,13,14,15], with Mimics being the most commonly used. Nevertheless, these software are operated by doctors with bare hands and rely on the surgeon’s experience and subjective judgment, making the process time-consuming and less reliable in terms of quality, efficiency, and consistency [16]. Therefore, efficient and intelligent automatic pedicle screw planning is becoming a research and application hotspot.

Early studies on automatic pedicle screw planning focused on anatomic shape optimization or template matching methods [17,18,19,20], which had poor accuracy and could not fully meet clinical needs, limiting their ability to simplify clinical work. The rapid development of artificial intelligence (AI) technology has propelled the intelligent progress in medicine. Among them, machine learning is an important method for achieving AI based on data learning through computer algorithms. In machine learning, neural networks can utilize various algorithmic model frameworks and have the ability to process complex tasks like the human brain, such as medical image diagnosis. Image segmentation is the foundational work of image diagnosis, a preparation for extracting more complex image features, and a prerequisite for developing more powerful diagnostic tools and generating surgical plans [21]. In 2021, Hallina et al. [22] used convolutional neural networks (CNN) to classify the narrowing of the central canal, lateral recess, and intervertebral foramina, with the overall classification accuracy of the model measured by the κ value being 0.82, 0.72, and 0.75, respectively; Zheng et al. [23] proposed a high-precision grading method for lumbar disc degeneration based on deep learning, with a prediction accuracy of 92.02%; using this indicator can better complete the grading task of lumbar disc degeneration. In 2022, Doerr et al. [24] applied the Faster R-CNN deep learning algorithm framework to realize the classification of thoracic and lumbar injuries on CT images, and the binary accuracy rate of determining injuries was 95.1%. In summary, compared with traditional CT images, AI can provide richer and more accurate data in the diagnosis of lumbar spine diseases. In addition, applying AI to preoperative planning can effectively reduce the difficulty of doctors’ decision-making and operational technical difficulty, improve the accuracy of intraoperative operations, and enhance surgical safety. Yamada et al. [25] trained AI to use a new algorithm to integrate the advantages of two types of images, and the generated 3D model can achieve more accurate preoperative planning, such as measuring the distance from the upper joint process to the exiting intervertebral foramen, to determine the puncture angle and position, and reduce the risk of intraoperative nerve damage or organ puncture. Currently, the application of deep learning in the planning of pedicle screws is booming [26,27,28]; however, research on automatic planning software for pedicle screws is limited, and its performance has not been fully evaluated. Existing deep learning algorithms still need further development and improvement.

In this study, we developed a CT image-based automatic pedicle screw planning software according to the self-developed deep learning algorithm, which can automatically segment the spine and plan the pedicle screw channels. Planning effectiveness and efficiency of pedicle screw implantation were evaluated quantitatively and qualitatively and compared with manually planned screws, considering variability between raters and planning software.

## 2. Materials and Methods

### 2.1. Patient Selection

Inclusion criteria: (1) from January 2016 to June 2023, patients with degenerative lumbar diseases (including lumbar stenosis, lumbar disc herniation, and degenerative lumbar spondylolisthesis) were treated; (2) patients underwent the first posterior lumbar fusion surgery (1–3 segments) at Beijing Chao-yang Hospital, Capital Medical University; (3) the above patients were treated with lumbar three-dimensional CT images at our hospital after conservative treatments failed.

Exclusion criteria: (1) previous history of lumbar surgery; (2) lumbar vertebrae with lateral curvature deformity; (3) patients with spinal tumors or pathological fractures. This study complied with the Declaration of Helsinki and was approved by the Ethics Committee of Beijing Chao-yang Hospital (ID: 2024-KE-346). Because of the retrospective study design, the Ethics Committee waived the requirement for informed consent.

### 2.2. Datasets and Preprocessing

**Figure 1:**
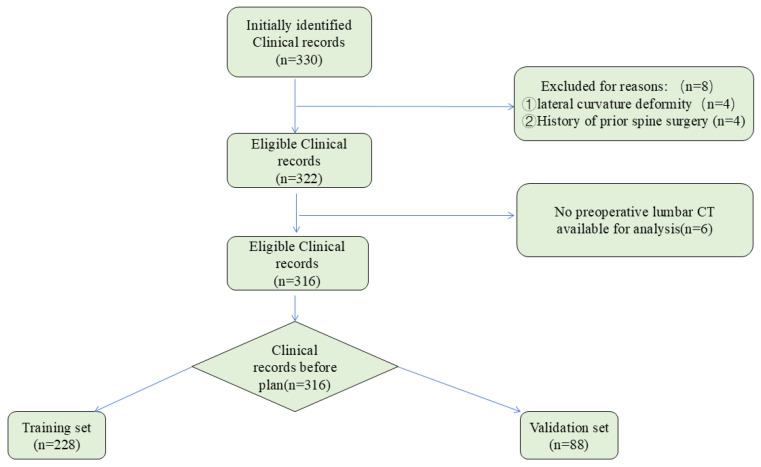
Flowchart describing the inclusion and analysis process.

In this investigation, we collected lumbar spine CT scans from various clinical databases that contained patients’ basic information, medical history, and imaging results. Among them, 316 patients (151 males and 165 females) were included, with an average age of (63.5 ± 9.3) years and an average BMI of (26.5 ± 4.0) Kg/m^2^. Among them, 80 cases were lumbar disc herniation, 153 cases were lumbar spinal stenosis, and 83 cases were lumbar spondylolisthesis. See Table 1 for details. To ensure accurate image processing, we adjusted the CT images for contrast and brightness to minimize effects from lighting variations and discrepancies in image acquisition settings. The analysis process is detailed in Figure 1.

### 2.3. Mimics Software Manual Pedicle Screws Preoperative Planning

Two experienced spin surgeons (Manual M1: B.D.W.; Manual M2: C.Y.Z.) manually planned the pedicle screw placements. Four months later, planner A performed a second planning session. The CT image data of lumbosacral vertebra were first imported into Mimics software 21.0 (Materialize, Leuven, Belgium). Based on the threshold method, we reconstructed the bone structure by adjusting the threshold settings. After selecting the target bone structure, threshold growth segmentation was performed, and then manual correction was performed. Two surgeons (M1 and M2) manually segmented the images, and a chief physician reviewed and confirmed the results. Any unqualified cases were revised until they passed the final review. We used manually segmented CT data as the standard reference (ground truth). Next, using the “Med CAD” function in Mimics software, we designed and drew a cylinder virtual pedicle screw, placed the screw according to the “^” shape crest, and properly adjusted the screw specifications and tracks, so that the virtual screw was in line with the axis of the pedicle, with an insertion depth of 80% of the length of the bone–screw channel. The screw’s diameter was designed to avoid penetrating the pedicle cortex, while maximizing the internal diameter. Finally, we measured the length, diameter, pedicle transverse angle (PTA), and pedicle sagittal angle (PSA).

### 2.4. Development and Construction of the Artificial Intelligence Preoperative Planning for Posterior Lumbar Interbody Fusion (AISPINE)

AISPINE (Version 1.0, Longwood Valley Technology, Beijing, China) consists of two modules: CT image processing and component planning. First, CT images were segmented to identify vertebral bodies, and data on screw positions and sizes were defined in the training set. The software then automatically identified the “entry point” and “exit point” of the implanted screws on the lumbar vertebra. The component planning module can plan the screw’s size, internal inclination, and head inclination; adjust its position in a 3D view; determine the state of the implant; and visualize the situation after placing the screw.

#### 2.4.1. Image Segmentation

**Figure 2:**
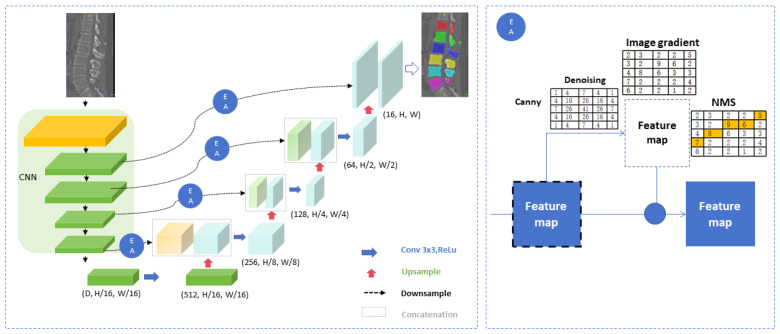
Development of artificial intelligence preoperative planning and patient-specific instrument systems for posterior lumbar interbody fusion (AISPINE). Network structure for image segmentation.

The complete dataset is randomly divided into to a training set and a validation set. First, CT images in DICOM format are converted to 3D.NIi.gz format. Before establishing the model, the window width and position of the CT images were adjusted. The window settings were a center of 150 and a window width of 1500. The neural network structure was developed based on 3D-UNet, which comprises two parts: encoding and decoding (as shown in Figure 2). The input CT images of the whole sequence are composed of four subsampling modules. Each module has two convolutional layers (3 × 3 × 3) and one max pooling layer, as well as an activation layer with ReLU as the activation function. Through subsampling in the encoding part, the model gradually extracts high-level semantic information from CT images. The decoding part has four upsampling modules. Each module includes two convolutional layers of 3 × 3 × 3, a deconvolution layer to increase feature map resolution, and an activation layer with ReLU. This upsampling helps the model better understand the low-level semantic information in the image. In the corresponding position of encode and decode, a skip connect module is designed to introduce an edge attention mechanism based on Canny algorithm, which is the edge attention (EA) module (Figure 2).

To evaluate neural network segmentation, the Dice coefficient [29] was calculated to measure the accuracy of vertebral body segmentation compared to manual segmentation. Also, the time taken for 3D-UNet segmentation of CT images was compared with that of manual segmentation.

#### 2.4.2. The Identification Model of Entry Point and Exit Point

**Figure 3:**
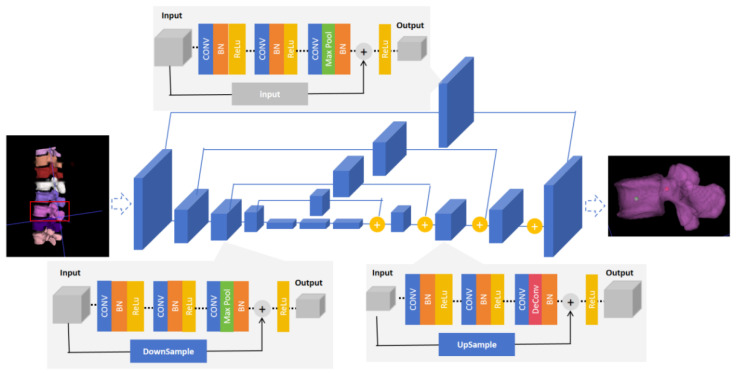
Identification of the Hourglass structure of the in-pin and out-pin points.

In this study, we used a 3D key-point recognition algorithm based on the Hourglass structure to detect the “entry point” and “exit point” for screw implantation in the lumbar vertebra. The core of the Hourglass network architecture is its symmetrical encoder–decoder design, the “hourglass” shape (as shown in the Figure 3). The network consists of multiple stacked Hourglass modules, each with a downsampling and an upsampling path. The downsampling path has four modules, and each module includes a 3 × 3 × 3 convolutional layer, a batch normal layer, and a ReLU activation function, forming a downsampling group. Each module consists of three subsampling groups, with a residual module added to the third group for improved model stability. The upsampling path has four upsampling models, each structured similarly to the downsampling module, including a convolutional layer, batch normal layer, and ReLU activation function. Moreover, a residual module is also inserted into the third group to improve the model’s overall stability. Finally, at the last layer of the upsampling path, the network outputs a set of heatmaps, each representing a key point. From the maximum positions in these heat maps, the entry and exit points of the nails implanted in each vertebra are predicted.

#### 2.4.3. Preoperative Planning Module

**Figure 4:**
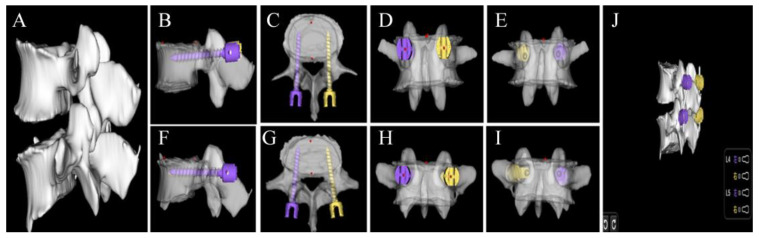
Posterior lumbar interbody fusion (AISPINE) preoperative screw planning module. (**A**) Three-dimensional reconstruction of L4 and L5; (**B**–**E**) preoperative planning of lumbar screws in L4; (**F**–**I**) preoperative planning of lumbar screws in L5; (**J**) postoperative implantation for 3D reconstruction.

A 3D model of the patient’s lumbar spine is created through CT segmentation and recognition. The size and angle of the screw can be adjusted in the coronal, sagittal, and transverse planes, with 80% of the bone–screw channel length set as the insertion depth. The screw’s diameter design avoids penetrating the pedicle bone cortex, while maximizing the use of the inner diameter of the vertebral arch root (Figure 4). Each adjustment allows real-time observation of screw placement and a 3D simulation of postoperative effects to select the optimal screw size, angle, and insertion point based on the surgeon’s preference. Finally, the dimensions and angles were recorded. Furthermore, the time required for automatic and manual pedicle screw implantation for each lumbosacral vertebra was recorded.

### 2.5. Quantitative Evaluation of Automatic Pedicle Planning

To assess the dimensions of pedicle screws, including length and diameter, as well as PTA and PSA, we will employ a validation method that involves comparing the automated planning of screws in the verification dataset with those planned manually, with the latter serving as the baseline truth. Additionally, to appraise the intra-rater reliability and the inter-rater agreement of manual screw planning, we will analyze and compare the screw plans established by two distinct raters, identified as M1 and M2.

### 2.6. Qualitative Evaluation of Automatic Pedicle Planning

In order to conduct qualitative validation, based on the Gertzbein–Robbins (GR) categorization, the AISPINE screw projections were appraised by two expert spinal surgeons, both with a wealth of experience exceeding ten years in the field of spinal fixation procedures (labeled as M3 and M4). In GR classification, pedicle screw placement relative to the cortical border was divided into cortical notch (grade A), <2 mm (grade B), <4 mm (grade C), <6 mm (grade D), and ≥6 mm (grade E). In addition, the direction of the pedicle perforation of all non-GR class A screws was recorded (medial, lateral, upper, and lower). In addition, Badu grading was used to evaluate the invasion of the upper articular process by the automatic planning screw: grade 0: the screw did not pass through the articular surface or invade the facet joint; grade 1: the screw is located on the side of the joint surface, but does not enter the facet joint; grade 2: the screw invades the joint ≤1 mm; grade 3: the screw passes through the joint surface.

### 2.7. Statistics

The quantitative validation of continuous variables was assessed through the calculation of their average values and the extent of variation from the mean, which is represented by the standard deviation. Shapiro–Wilk normal test was used for the distribution of values, and ANOVA was used for non-parametric test. For matched data, the Friedman test was performed, and followed by Dunn’s multiple comparison test to compare results between automatic and manual scheduling screws. The Wilcoxon paired non-parametric test compares the planning time of both methods. *p* < 0.05 was considered statistically significant. The chart shows violin-like structures in the 5 to 95% percentiles. Statistical analysis was performed using GraphPad Prism v9.0.

## 3. Results

### 3.1. Descriptive Data

Of the 330 patients, 8 patients were excluded due to lateral curvature deformity (*n* = 4) and a history of prior spinal surgery (*n* = 4); furthermore, 6 patients were excluded due to missing preoperative lumbar CT scans. Thus, 316 clinical records met the inclusion and exclusion criteria. Patient details are summarized in Table 1.

The dataset for training encompassed 1090 screws across 228 distinct cases, spanning from the L2 to S1 spinal levels. For the validation series, 438 screws from 88 cases were considered, with specific distributions at L2 (4 screws), L3 (48 screws), L4 (164 screws), L5 (168 screws), and S1 (54 screws). The median number of spinal levels involved in the construct was one, with a spread from one to three levels. The automated planning protocol was successfully implemented for the entire set of 438 screws.

**Figure 5:**
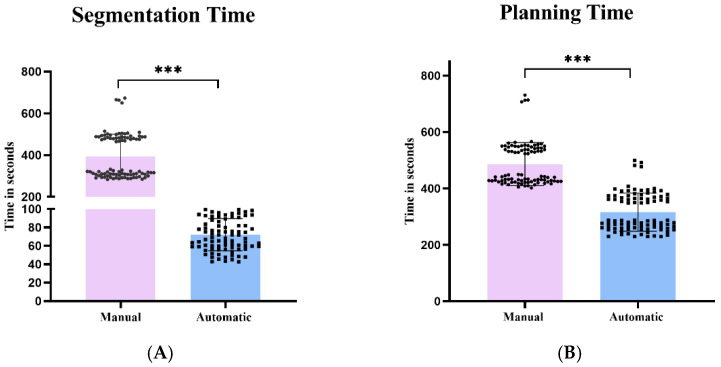
Manual and automatic time schedule. (**A**) Examination of the duration needed for automated and manual segmentation tasks of the lumbosacral spine, measured in seconds per screw; (**B**) examination of the duration needed for automated and manual planning tasks of the lumbosacral spine, measured in seconds per screw. Utilizing the Wilcoxon test for dependent pairs.*** *p* < 0.001.

The Dice segmentation coefficients of lumbosacral vertebrae were as follows: 0.9533 for L2, 0.9518 for L3, 0.9506 for L4, 0.9481 for L5, and 0.9438 for sacral 1, with an overall Dice coefficient of 0.9459 (Table 2). Manual segmentation time is 393.91 ± 107.10 s, while automatic segmentation time is 72.10 ± 17.51 s (Figure 5A).

### 3.2. Quantitative Evaluation of AISPINE Screw Plans

**Figure 6:**
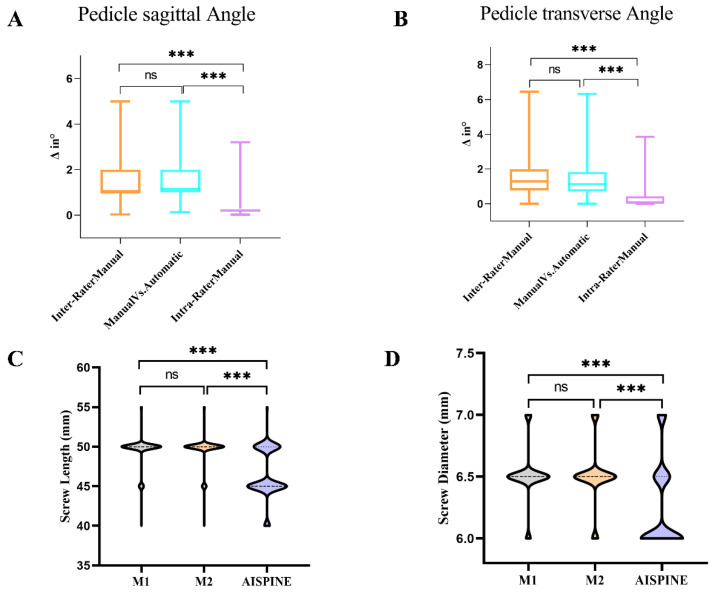
(**A**–**D**) Results from quantitative evaluation. (**A**) The PSA results of automatic screw planning were compared with those of manual screw planning for reference. (**B**) The PSA results of automatic screw planning were compared with those of manual screw planning for reference. The automatically planned screw length (**C**) and diameter (**D**) are used as reference to compare with the manually planned screw length (**C**) and diameter (**D**). The Friedman test was statistically significant, followed by Dunn’s multiple comparisons. *** *p* < 0.001. ns represents no significance.

The results of automatic screw planning were compared with those of manual planning as the underlying truth, and the background of the differences is determined by comparing the inter-variance and intra-variance of manual planning screws. The quantitative verification results of the planning algorithm are shown in Figure 6.

The screw orientation of PSA evaluation showed an average deviation of 1.63 ± 0.83° between automatic and manually planned screws, which was similar to the inter-group variance of 1.54 ± 0.84°. However, it was significantly higher than the intra-group variance of 0.39 ± 0.49° (*p* < 0.001) (Figure 6A). In addition, the screw orientation of TSA evaluation showed an average deviation of 1.39 ± 1.03° between automatic and manually planned screws, which was similar to the inter-group variance of 1.46 ± 0.99°. However, it was significantly higher than the intra-group variance of 0.30 ± 0.48° (*p* < 0.001) (Figure 6B).

The mean screw length for automatic scheduling screws planning was 46.2 ± 3.6 mm and 49.5 ± 4.1 mm for manual scheduling screws, indicating that automatic scheduling screws were shorter and statistically significant (*p* < 0.001) (Figure 6C). Average screw diameters for automatic planning screws ranged from 6.24 ± 0.35 mm, and 6.51 ± 0.22 mm for manual planning screws, indicating that automatic planning screws were thinner and statistically significant (*p* < 0.001) (Figure 6D). There was no significant variance difference between M1 and M2 in manual planning screw lengths and diameters.

**Figure 7:**
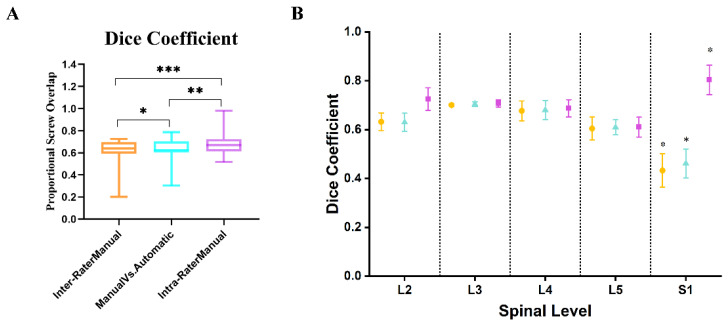
The Dice coefficient of the corresponding screw overlap. (**A**) Represents the overlap proportion of the whole screw, (**B**) represents the overlap proportion of each spinal level screw. Circle (○) represents inter-variance manual planning; the triangle (△) indicates automatic vs. manual planning; square (□) displays intra-variance manual planning; symbols show mean ± SD. * *p* < 0.05. ** *p* < 0.01. *** *p* < 0.001.

The Dice coefficient of the automatic screw and the manual screw indicates that the proportional overlap of the corresponding screw plan is 0.63 ± 0.08. This showed significantly better spatial matching compared to manual planning screws, with a variance of 0.62 ± 0.09 (*p* < 0.0001). (Figure 7A).

The average time required for automatic screw planning was 316.08 ± 68.17 s, compared to 486.40 ± 77.24 s for manual planning. As a result, the automatic screw planning is faster (*p* < 0.0001) (Figure 5B).

Furthermore, we utilized the Dice similarity coefficient to evaluate the agreement of screw planning at different spinal levels (Figure 7B). There was no significant discrepancy in agreement among all groups (automated versus manual, inter-variability, and intra-variability) at the L2 and L5 levels. Nevertheless, the Dice coefficients for the S1 level were significantly lower in all groups, suggesting higher variability in the placement of screws at this level, which is indicative of the less dense bone anatomy of S1 as compared to other spinal levels (*p* < 0.001).

### 3.3. Qualitative Evaluation of AIPSINE Screw Plans

**Figure 8:**
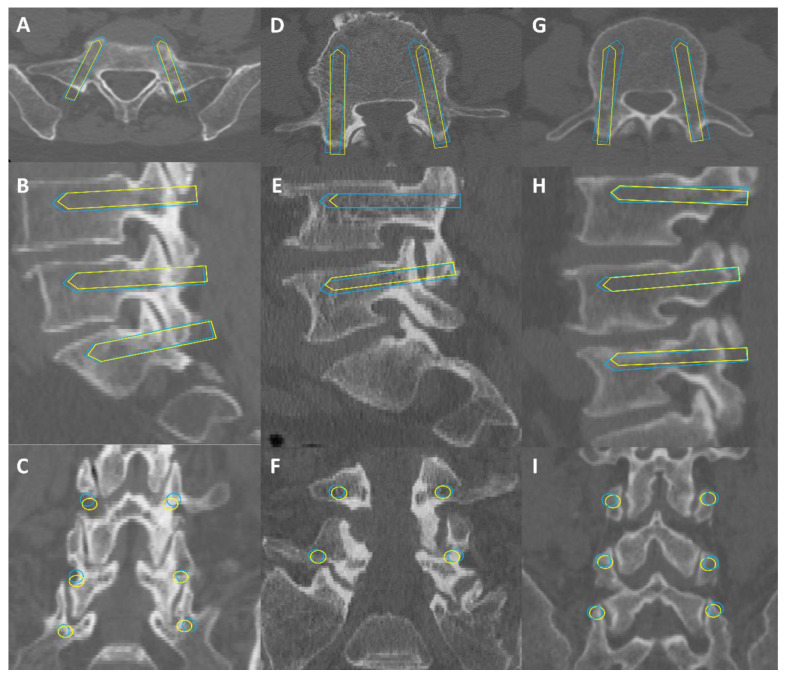
Illustrative cases. Illustrative cases depicting close agreement of automatically planned lumbar pedicle screws (blue) and their corresponding manually planned reference (yellow) in axial, sagittal, and coronal reconstructions, respectively. Case 1 (**A**–**C**) describes two-level fusion of degenerative spondylolisthesis. Case 2 (**D**–**F**) shows a single-level fusion of a lumbar disc herniation. Case 3 (**G**–**I**) indicates the fusion of two levels of lumbar spinal stenosis.

Screw fit in the pedicle was assessed using Gertzbein–Robbins grading. All manually planned screws achieved GR grade A, which provided reference for this study. AISPINE produced 428 GR-A screws (97.7%), while 10 screws (2.3%) were GR grade B, showing mild cortical rupture <2 mm, all affecting the L5 segment. Badu grading assessed damage to facet joints, with 426 screws (97.3%) at Badu level 0 and 12 screws (2.7%) at Badu level 1, all also affecting the L5 segment. Table 3 summarizes the qualitative evaluation of screw planning, and Figure 8 shows three cases illustrating screw planning accuracy evaluated in this study.

## 4. Discussion

Pedicle screw placement is crucial for successful PLIF surgery. Detailed preoperative planning can enhance success rates, reduce radiation exposure, and shorten surgery time. However, manual screw planning software, such as Mimics, is still time-consuming, and current proposed methods for automating screw planning have limitations that affect clinical applicability and accuracy [17,18,19,20]. This motivated us to develop and validate a software tool to simplify screw planning to better assist in lumbosacral internal fixation surgery. After training, the deep learning–based algorithm, using existing real CT data, shows strong correspondence in quantitative and qualitative validation. Automatic planning with manual screws and automatic planning results showed clinically acceptable accuracy. Compared to the scoring differences of manual screw planning, our algorithm is no less effective than manual scoring, but it is more time-efficient.

### 4.1. Data Processing

Accurate CT data segmentation is essential for PLIF preoperative planning and can provide reference for pedicle screw placement. In this study, manual planning with Mimics software, involving extensive corrections by experienced physicians, achieved good clinical results. However, this process is time-consuming and labor-intensive, impacting the efficiency of CT-based preoperative planning. With advancements in AI and deep learning, automatic segmentation of medical images has become more efficient [30,31,32]. This study uses 3D-UNet convolutional neural networks; the key innovation of 3D-UNet is that it uses 3D convolutional operations, which enables the network to capture both spatial depth and contextual information. This feature makes it highly effective for 3D medical image segmentation tasks, enabling precise identification and segmentation of complex anatomical structures. The convolutional neural network segmentation model treats each patient’s CT image as an array unit that can process multiple layers of data simultaneously. Manual segmentation requires layer-by-layer segmentation, which is less efficient [33]. In addition, we introduce a Canny algorithm-based EA mechanism [34], to enhance the algorithm’s ability to recognize the vertebral body edges, and we can better retain the edge and detail information of the image in the decoder stage, thus improving segmentation accuracy. In our study, we utilized the training data with 228 lumbar CT images, to manually cut corresponding surgical segments. We used the deep learning algorithm framework based on 3D-UNet+EA to derive a convolutional neural network model capable of automatically segmenting lumbar vertebrae, and used 88 independent verification sets of lumbar CT images to verify the model. The results show that the overall Dice coefficient of automatic segmentation was 0.9459, and the automatic segmentation time was only one-fifth of that of manual segmentation, which was superior to similar segmentation results based on lumbar CT [35]. Therefore, we conclude our 3D-UNet+EA algorithm significantly improves both the speed and accuracy of lumbosacral segmentation.

### 4.2. Quantitative Validation

For PLIF patients, proper screw size and trajectory are crucial for postoperative function and long-term survival. Many studies have aimed to find optimal screw placement guidelines [36,37], but a universally accepted standard has not been established yet. Despite acceptable appearances on postoperative X-ray images, there remains inherent variability between screws that is considered to be within tolerable limits [7]. Individual differences in bone structure and lesions highlight the need for individualized screw planning. Early studies on automated screw channel planning concentrated largely on introducing prior case knowledge to calculate the optimal pedicle screw channel [17,18,38], but most of these methods rely on manual intervention to set the initial parameters, leading to lower accuracy and poor robustness. In this study, we used a 3D key-point recognition algorithm based on the Hourglass structure to identify the “entry point” and “exit point” for screws in the lumbar vertebra. The Hourglass network is a multi-scale convolutional neural network commonly used in human pose estimation and critical point detection tasks [39]. Its multi-scale nature and contextual information fusion capability make it suitable for processing complex 3D images. Instead of directly comparing automatic planning screws with the corresponding reference screws, we evaluated our results by comparing them with screws from manual planning methods M1 and M2. Moreover, supplementary qualitative assessments guarantee that the screws are clinically acceptable, notwithstanding the quantitative differences that may be observed.

Accurately determining the direction, encompassing PSA and PTA, is the initial step in establishing a pedicle screw channel. The research findings demonstrate that the intra-variability among two distinct manual planners is markedly greater than the intra-variability observed when the same manual planner conducts the screw planning on two separate occasions (Figure 6). This suggests that despite the presence of defined guidelines and anatomical limitations, each surgeon tends to evolve a personalized approach to screw planning.

Our quantified evaluation reveals that the results from automatic screw planning are within the variance range among different planners as well as within the same planner, signifying that the training of the algorithm is not swayed by the individual traits of a single planner. Most crucially, the variance in screw positioning between automatic and manual planning was either similar to, or significantly lower than, the variance between manual planners (Figure 6). Moreover, the Dice coefficient demonstrated that the congruence between screws planned automatically and those planned manually was statistically higher than the consistency between two independent manual evaluations of screws. Additionally, the automatic planning of screws not only upheld non-inferiority but also considerably enhanced efficiency in comparison to manual planning (Figure 5). In summary, these discoveries suggest that the performance of automatically planned screws excels that of manually planned screws.

We found that sacral screws had much more variability compared to lumbar screws, with no significant difference between manual and automatic planning at the lumbar level (Figure 7). This is because the sacrum’s anatomy allows for more freedom in cranial angles and screw convergence than in other vertebral segments of the lumbar or thoracic vertebrae [7].

In terms of screw length and diameter, auto-planned screws are shorter and slightly thinner than manually planned screws. Because the AISPINE software sets strict boundary conditions, if the screw is a little longer or thicker, it will touch the boundary conditions we set, and the system will automatically select the screw that is a little shorter and thinner. In addition, even if the automatically planned screws are shorter and thinner, the error with the gold standard manually planned screws is within the acceptable range. The automatic planning of the selection of thinner and shorter screws can provide the surgeon with an initial reference, and the specific adjustment can be fine-tuned according to the specific intraoperative situation. Compared with manual planning of selected screws, it is more convenient, less time-consuming, and the accuracy is no less than manual planning.

### 4.3. Qualitative Validation

GR classification is often used to evaluate pedicle screws based on their position within the cortical boundary [40]. In this study, we used deep learning methods to learn screw positioning from the training set data, and verified that 97.7% of patients in the set achieved grade A, and only 2.3% of patients had minor and clinically acceptable fractures (grade B), similar to the results of previous studies [41,42]. In addition, in this study, we evaluated screw invasion of facet joints according to Badu grading and found that 12 screws (2.7%) had such invasion, which was lower than that in similar studies [26]. Screw invasion of the upper articular process can change the stress distribution, leading to spontaneous fusion of the articular process and adjacent vertebral lesions, which should be avoided [43]. The articular process invasion occurred at L5, which was mainly considered to be related to the training data. In order to facilitate the insertion angle of L5, the human point was intentionally tilted inward toward the articular process during planning, and the training learning based on these data may result in sub-optimal screw positions.

The deep learning platform proposed in this study can be integrated into clinical diagnostic workflows. Firstly, lumbar CT image data were collected, which required strict pre-processing, including denoising, standardization, and enhancement. Then, the 3 U-Net+EA segmentation model developed by us was used to accurately segment and identify the lumbar vertebra in the image. First proposed by Cicek et al. in 2016, 3D U-Net is a deep learning model used to solve the segmentation problem in 3D medical images, such as the processing of CT, MRI, and other images [44], and is an extension of 2D U-Net with a symmetrical encoder–decoder structure. The encoder part is responsible for gradually extracting the context information of the image and reducing the size of the feature map through convolution and subsampling layers. In the decoder part, the spatial resolution of the feature image is gradually restored through deconvolution and the upper sampling layer to generate high-resolution segmentation results. Our experimental results show that 3D U-Net exhibits high precision and robustness in the task of segmentation of the lumbar vertebra, which further proves its potential in 3D medical image segmentation. In addition, we introduced an edge attention mechanism based on Canny algorithm in the skip connection module of 3D U-Net. Specifically, we apply Canny edge detection to the feature map generated by each encoder to extract the edge information of the image. This edge information is used to enhance feature maps in skip connection to highlight important edge features during decoding, significantly improving segmentation accuracy, especially in complex anatomical structures such as the lumbar vertebra region. Next, the trained deep learning model is integrated into the existing clinical diagnostic workflow, which may involve the development of interfaces with the hospital information system (HIS) and the image storage and transmission system (PACS) to achieve data flow and processing. Finally, the deep learning model is applied in the actual clinical environment to assist doctors in the diagnosis of lumbar diseases. At the same time, feedback from physicians needs to be collected to continuously optimize the performance of the model.

### 4.4. Limitations

This study was a preliminary exploration of automatic pedicle planning, which had some limitations: (1) screw selection was not combined with bone density and biomechanical analysis; (2) the screw trajectory was not evaluated in combination with the distance and angular deviation between entry and exit points, because the system lacked 3D spatial coordinates. However, we compared PTA and PSA between the two planning methods, which provided a reference for evaluating the pedicle trajectory; (3) only lumbosacral vertebrae data were calculated in this study, and thoracic and cervical vertebrae segments need to be further verified.

Deep learning technology shows great promise but faces several challenges, including a lack of a large amount of data to train, which can lead to bias, large costs of manual labeling and planning, and lack of relevant evaluation criteria [45]. In addition, after the proof-of-concept of automatic screw planning using the 3D-UNet+EA segmentation algorithm and Hourglass is provided in this study, to optimize this performance, it is necessary to incorporate additional training data tailored to specific pathological states, and independent validation will demand a more detailed assessment procedure.

All the data in this study were from the same CT scanner, and the compatibility of the algorithm with other scanners has not been systematically evaluated. However, since CT grayscale values are based on standardized Horsfield units, this usually helps maintain the algorithm’s accuracy across different CT scanners. In future work, further external validation is needed to confirm our results.

## 5. Conclusions

Within the scope of this research, we developed and verified AISPINE, a software application that employs deep learning methodologies for the complete automation of segmentation and planning of lumbosacral pedicle screws. The results show that it performs as well as manual planning, providing accurate and faster screw implantation process for preoperative planning. These results offer great potential for improving the workflow of spinal surgery.

## Figures and Tables

**Table 1:** Descriptive data.

Variable	Patients in Test Set (*n* = 88)	All Patients (*n* = 316)
Age, years	62.6 ± 10.3	63.5 ± 9.3
Gender, *n*		
Male	40 (45.5%)	151 (47.9%)
Female	48 (54.5%)	165 (52.1%)
BMI, Kg/m^2^	26.5 ± 4.2	26.5 ± 4.0
Diagnosis, *n*		
Lumbar disc herniation	31 (35.2%)	80 (25.3%)
Lumbar spinal stenosis	38 (43.2%)	153 (48.4%)
Lumbar spondylolisthesis	19 (21.6%)	83 (26.3%)
Construct length in spinal segments, *n*		
1	49	199 (63.0%)
2	35	102 (32.3%)
3	4	15 (4.7%)
Screws evaluated per spinal segment, *n*		
L2	4 (0.9%)	30 (2.0%)
L3	48 (11.0%)	176 (11.5%)
L4	164 (37.4%)	534 (35.0%)
L5	168 (38.4%)	604 (39.5%)
S1	54 (12.3%)	184 (12%)

Evaluation of AISPINE lumbosacral vertebral segmentation accuracy.

**Table 2:** Comparison of accuracy and consistency of lumbosacral vertebrae segmentation.

	L2	L3	L4	L5	S1	Total
Dice	0.9533	0.9518	0.9506	0.9481	0.9438	0.9459

**Table 3:** Qualitative screw evaluation.

N = 438 Screws	GR-Grade A	Non GR-Grade A	Badu 0	Non Badu 0
Manual planning	* 438 (100%)	0	438 (100%)	0
AISPINE planning	428 (97.7%)	10 (2.3%)	426 (97.3%)	12 (2.7%)

GR Gertzbein–Robbins grade. * Reference screws were planned independently by two experts in spine surgery.

## Data Availability

Access to the dataset shall be provided by the corresponding authors upon reasonable request and in accordance with the policies of the relevant institution.
